# Supplementary material for: Comprehensive Annotation and Functional Exploration of MicroRNAs in Lettuce
Source: Front Plant Sci. 2021 Dec 24;12:781836. doi: 10.3389/fpls.2021.781836 (PMC8739914; doi:10.3389/fpls.2021.781836)
Supplement: Supplementary file 3 [file Data_Sheet_9.ZIP › GSM2230752.plot/Lsa-miRN1698_Lsat_1_v5_gn_1_82460.1_415_TPlot.pdf]

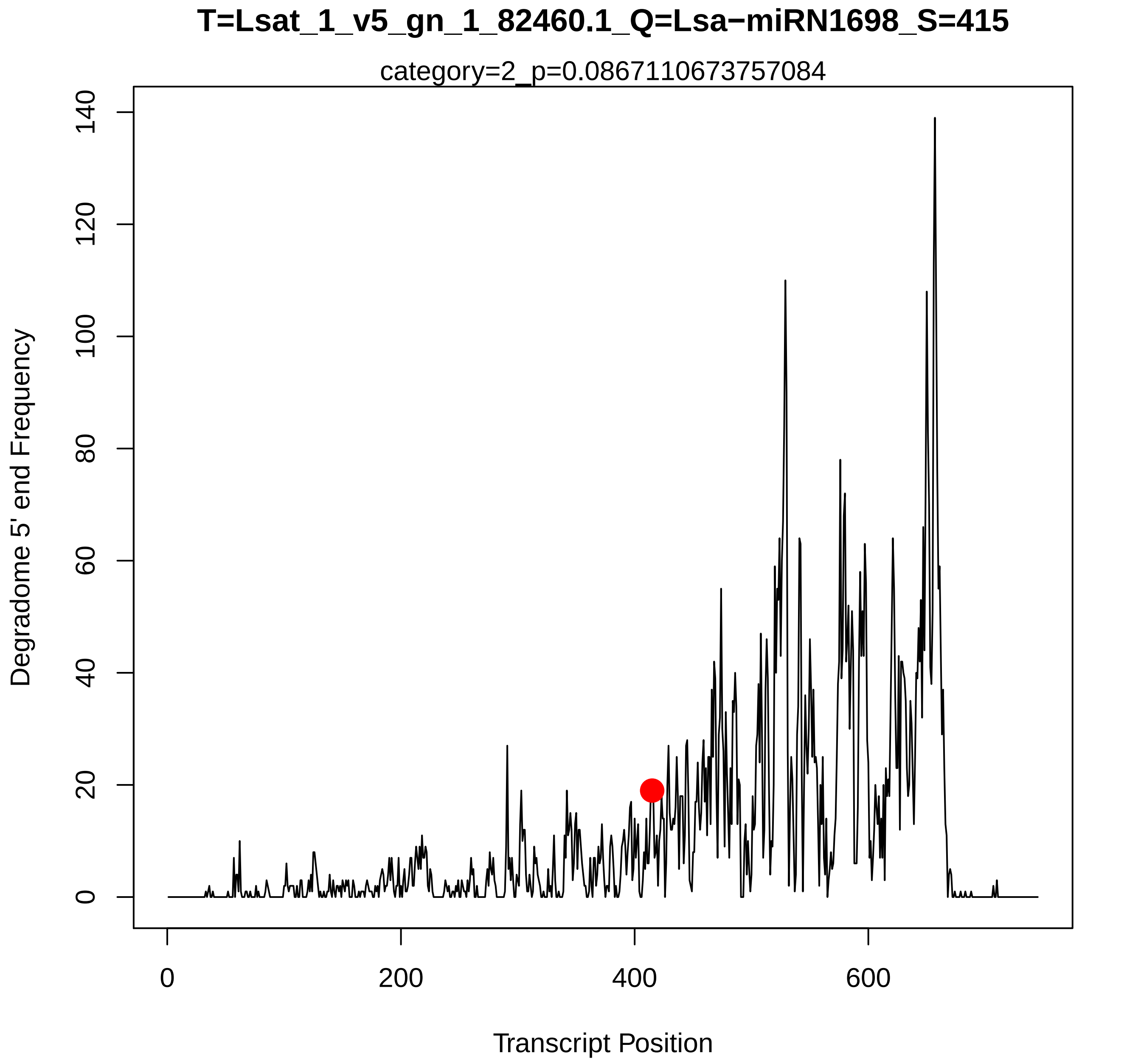
T=Lsat_1_v5_gn_1_82460.1_Q=Lsa−miRN1698_S=415
category=2_p=0.0867110673757084
Degradome 5' end Frequency
0
20
40
60
80
100
120
140
0
200
400
600
Transcript Position
